# Clinical features and prognosis of patients with thrombotic thrombocytopenic purpura associated with systemic lupus erythematosus: a review of 25 cases

**DOI:** 10.1186/s13052-019-0641-y

**Published:** 2019-04-29

**Authors:** Ji Li, Jing-Jing Jiang, Chang-Yan Wang, Shan Jian, Yu Zhou, Ming-Sheng Ma, Xiao-Yan Tang, Lin Wang, Mei-Ying Quan, Yu Zhang, Juan Xiao, Yan-Yan He, Hong-Mei Song

**Affiliations:** Department of Pediatrics, Peking Union Medical College Hospital, Chinese Academy of Medical Sciences, No. 1 Shuaifuyuan, Dongcheng District, Beijing, 100730 China

**Keywords:** Thrombotic thrombocytopenic purpura, Systemic lupus erythematosus, Clinical features, Prognosis

## Abstract

**Objective:**

To report the clinical features of patients with systemic lupus erythematosus (SLE) associated with thrombotic thrombocytopenic purpura (TTP). Their diagnosis, treatment, and prognosis were also discussed.

**Methods:**

A total of 25 TTP-SLE pediatric patients were included in this study. Their clinical symptoms, laboratory findings, disease activity, and renal biopsy were retrospectively reviewed.

**Results:**

The median age of the patient cohort was 14 years old. Nine patients were first diagnosed with SLE, followed by the diagnosis of TTP-SLE, whereas 15 patients were diagnosed with TTP and SLE concurrently. All the 25 TTP-SLE patients had decreased platelet count and microangiopathic hemolytic anemia. Fever, rash, edema and neurological symptoms were the main clinical symptoms. Fragmentation of erythrocytes on blood smear and increased LDH were found in all patients. Nineteen patients (76%) had impaired renal function. Renal biopsy showed that most of the patients had lupus nephritis class IV (20%) and TMA (20%). 13 patients (52%) were treated with glucocorticoids in combination with immunosuppressive agent, and 10 patients (40%) were treated with plasma exchange combined with glucocorticoids plus immunosuppressive agent. One patient died due to lung infection; others had disease remission. Fifteen patients had follow-up regularly, and their conditions were stable.

**Conclusion:**

Patients with TTP-SLE often had moderate to severe lupus disease activity. Testing of LDH level and blood smear should be performed when kidney and neurological symptoms arise in children with SLE. The use of combination therapy, glucocorticoids plus immunosuppressive agent, provided satisfactory clinical outcome. Patients with refractory TTP-SLE will also need plasma exchange therapy.

## Background

Thrombotic thrombocytopenic purpura (TTP) is a disorder of the blood-coagulation system. It is characterized by small blood clots formed throughout the body, resulting in thrombocytopenia. The condition can result in damage to major organs or death. TTP can be divided into acute idiopathic TTP and secondary TTP, which has been seen in association with malignancy, infections, and drugs such as ticlopidine, cyclosporine, and mitomycin C [1, 2]. Although TTP in patients with systemic lupus erythematosis (SLE) is rare, TTP-SLE has high mortality, ranging from 34 to 62.5% [3, 4].

The pathogenesis of TTP-SLE is related to endothelial injury or platelet aggregation that lead to vascular injury or autoimmune response [5, 6]. Patients with TTP were found to have unusually large von Willebrand factor (VWF) multimers and thrombi that were rich in platelet aggregates [3, 7]. Subsequent studies showed that the unusually large VWF multimers were hyperactive in binding and aggregating platelets [6]. ADAMTS13 (A Disintegrin and Metalloproteinase with ThromboSpondin-1-like motifs, member 13 of this family of metalloprotease)) is an enzyme that cleaves the adhesive VWF. The lack of ADAMST13 and the formation of large multimers of VWF can trigger intravascular platelet aggregation and microthrombosis, causing the signs and symptoms of TTP [6]. In addition, excessive hemoglobin and increased thrombin and D-dimer may also contribute to the development of thrombotic microangiopathies in TTP [8].

To date, treatment options for TTP-SLE patients include plasma exchange, administration of glucocorticoids, or immunosuppressive agents. Studies reported the presence of anti-endothelial cell antibodies, anti-platelet antibodies, and anti-ADAMTS13 antibodies in TTP-SLE patients [9, 10], implicating the role of autoimmune mechanism in pathogenesis of TTP-SLE. Rituximab is a monoclonal anti-CD20 antibody, which has demonstrated effectiveness in various autoimmune hematological disorders including idiopathic TTP [11].

In this study, we retrospectively reviewed the clinical and laboratory features of 25 children diagnosed with TTP-SLE. Their clinical manifestations, treatments, and prognosis were discussed.

## Patients and methods

### Patients

Clinical data of 25 children diagnosed with TTP-SLE in Beijing Union Medical College Hospital between May 2007 and February 2018 were reviewed and analyzed for the purpose of the study. The clinical data included laboratory findings, renal pathological damages, presentation of immune markers, and symptoms.

### Diagnostic criteria

SLE was diagnosed in accordance to the criteria defined by American College of Rheumatology (ACR) in 1997 [12].

TTP was diagnosed by the following criteria: 1) presence of microangiopathic hemolytic anemia (MAHA), in accompany with an increase of LDH value and fragmentation of erythrocytes on peripheral blood smear; 2) thrombocytopenia in the absence of other known causes; 3) with or without renal or neurologic abnormalities, or thrombotic microangiopathy (TMA) showed by renal biopsy.

### Statistical analyses

Statistical analysis was performed using SPSS 19.0 software. The continuous data were expressed as median (interquartile range), while the categorical data were expressed as frequency (%). Wilcoxon rank sum test was used to compare differences between groups. The *p*-value < 0.05 was considered as statistically significant.

## Results

### Epidemiologic characteristics

Among the 25 children with TTP-SLE, 8 were male (32%) and 17 were female (68%). The age of the disease onset ranged from 9 to 18 years old, with the median at 14 years old. In this patient cohort, 9 patients were diagnosed with SLE first, followed by TTP-SLE (36%). One patient was diagnosed with TTP first, followed by TTP-SLE (4%). Fifteen patients were diagnosed with TTP-SLE concurrently (60%). The time interval between the diagnosis of SLE and TTP ranged from 1 to 3 years, and these patients received treatment right after diagnosis of SLE. The average score of SLE disease activity (SLEDAI) was 22.4 points; 3 cases (12%) had moderate disease activity, and 22 cases had severe disease activity (88%).

### Clinical features

All the 25 TTP-SLE patients had decreased platelet count and microangiopathic hemolytic anemia. Fever (*n* = 20, 80%) was the most common clinical symptom observed, followed by rash (*n* = 10, 40%) and gastrointestinal symptoms (n = 10, 40%). Other clinical presentations include edema (*n* = 9, 36%), nervous system symptoms (n = 9, 36%), and joint pain (*n* = 5, 20%) (Table 1). Blood smear and Coombs test were performed in 17 patients; all of these patients had fragmentation of erythrocytes and 4 of them had Coombs positive. The increase of LDH was found in all patients (median = 528 U/L, range 265–2511 U/L). Nineteen patients (76%) had impaired renal function. The median of serum creatinine level was 115 umol/L (range 38–366.8 umol/L).

**Table 1:** Clinical manifestations in 25 patients

Clinical manifestation	Total cases *n* (%)	Clinical presentation	Total cases *n* (%)
Weakness	3 (12%)	Neurological system	9 (36%)
Fever	20 (80%)	Headache	5 (25%)
Rash	10 (40%)	Conscious disturbance	6 (24%)
Gastrointestinal system	10 (40%)	Seizures	6 (24%)
Diarrhea	1 (4%)	Language disorder	1 (4%)
Abdominal pain	3 (12%)	Hemiplegia	2 (8%)
Abdominal bloating	3 (12%)	Joint pain	5 (20%)
Vomit	5 (20%)	Urinary system	10 (40%)
Respiratory system	7 (28%)	Edema	9 (36%)
Chest pain	1 (4%)	Dark urine	2 (8%)
Dyspnea	6 (24%)		

The presentation of markers was also examined in all patients. More than 64% of children had presentation of the lupus-related markers, such as positive ds-DNA, low level of serum C3 or C4, and increased ESR. ADAMTS13 activity was tested in 3 patients, and 2 of them were positive (Table 2). Renal biopsies were performed in 10 patients, of which 8 patients showed renal pathological damage. Class IV (20%) and TMA (20%) were more frequently observed. Other types of damage include: class III (10%), class V (10%), class VI and TMA (10%), class IV and TMA (10%), class III and TMA (10%) (Table 3).

**Table 2:** Presentation of Immune Markers (*n* = 25)

Immune Markers	Total cases *n* (%)
Positive ds-DNA	16 (64%)
Low C3 (< 0.73)	20 (80%)
Low C4 (< 0.1)	20 (80%)
Erythrocyte sedimentation rate (ESR) > 15	22 (88%)
Positive ADAMTS13 activity^a^	2 (66.6%)

^a^ADAMT13 activity was tested in 3 patients only

**Table 3:** Renal pathological damages in 10 patients

Renal pathological damages	Total cases *n* = 10
III	1 (10%)
IV	2 (20%)
V	1 (10%)
IV and V	1 (10%)
VI and TMA	1 (10%)
III and TMA	1 (10%)
TMA	2 (20%)

*TMA: thrombotic microangiopathy

### Treatment and response to treatment

Combination therapies were widely used in patients with TTP-SLE. In this study, 13 patients (52%) were treated with glucocorticoids in combination with immunosuppressive agent, and 10 patients (40%) were treated with plasma exchange combined with glucocorticoids plus immunosuppressive agent. Cyclophosphamide was given for all patients received immunosuppressive agent. Mycophenolate mofetile was given as a maintenance therapy after cyclophosphamide treatment in 16 patients (15.2%). In addition, cyclosporine (*n* = 8, 7.6%), leflunomide (*n* = 1, 4%), and tacrolimus (*n* = 1, 4%) were also given. One patient was given with plasma exchange (7 times) in combination with glucocorticoids plus immunosuppressive agent. However, hemorrhagic cystitis and myelosuppression were observed upon the addition of low dose of cyclophosphamide, and the therapy was discontinued. Rituximab was then given to the patient, and the condition was relieved. A total of 11 patients underwent plasma exchange (44%), with median at 8 times. In addition, one patient got better with plasma disease (4 times) combined with glucocorticoid treatment. The patients were divided into three groups according to the treatment option given, and the laboratory parameters before and after treatment were compared between the three groups (Table 4).

**Table 4:** Laboratory findings before and after treatment

Treatment option	Laboratory parameter	Before treatment	After treatment
Plasma exchange + glucocorticoids + immunosuppressive agent (*n* = 10)	Leukocyte	3.41 (3.54)	6.28 (4.99)
	Platelet	40 (54)	132.5 (86.25)
	Hemoglobin	62.5 (26.25)	88.0 (15.25)
	LDH	528 (386)	286 (78)
	Creatinine	115 (136)	78 (67)
	Urea	17.3 (21)	10.69 (14.6)
Glucocorticoids + immunosuppressive agent (*n* = 13)	Leukocyte	6.97 (7.07)	6.95 (4.54)
	Platelet	38 (53.25)	159 (36)
	Hemoglobin	71 (36.35)	106 (45.75)
	LDH	541 (300)	242 (233)
	Creatinine	187 (133)	83 (85)
	Urea	17.94 (5.97)	7.1 (13.85)
Plasma exchange^a^ + glucocorticoids (*n* = 1)	Leukocyte	2.42	6.55
	Platelet	61	279
	Hemoglobin	81	131
	LDH	691	296
	Creatinine	407	279
	Urea	32.5	15.7
Plasma exchange + glucocorticoids + immunosuppressive agent + Rituximab (*n* = 1)	Leukocyte	2.7	3.5
	Platelet	59	108
	Hemoglobin	85	74
	LDH	619	246
	Creatinine	407	279
	Urea	38.2	27.7

^a^Plasma exchange was performed four times

### Prognosis

Of the 25 patients, 1 patient died due to fetal lung infection, and all the others had disease remission. Fifteen patients had regular follow-ups, and their conditions were stable.

## Discussion

The diagnosis of TTP in the setting of SLE may be challenging, as the diseases have overlapping clinical symptoms such as fatigue, rash, and fever. In this retrospective study, we found the neurological symptoms (e.g. headache, conscious disturbance), gastrointestinal symptoms (e.g. vomit, abdominal pain), and urinary symptoms (e.g. edema, dark urine) favored the diagnosis of TTP-SLE. Therefore, we suggested that when new kidney and neurological symptoms arise in children with SLE, testing of LDH level and blood smear should be done to allow early diagnosis of TTP-SLE. The rate of diagnosis of TTP and SLE simultaneously appeared vary among different geographic regions. A previous study in US reported that 73% of patients had SLE diagnosed before TTP onset, whereas only 15% of patients had SLE and TTP diagnosed simultaneously [3]. In the current study, 60% of patients were diagnosed with TTP-SLE simultaneously. The incident rate was similar to those reported locally. Studies suggested that patients with TTP-SLE often had moderate to severe lupus disease activity [13], and this is in accordance with the data we observed in this study (average SLEDAI = 22.4).

The MAHA in TTP and autoimmune hemolytic anemia (AIHA) in SLE have overlapping features; however, the diseases may be differentiated through blood smear and Coombs’ test. MAHA is manifested by erythrocyte fragmentation on peripheral blood smear, whereas AIHA is characterized by the presence of autoantibodies directed toward red blood cells, usually demonstrated by a positive Coombs’ test. In our study, 17 children completed the blood smear and Coombs’ test. All of them showed erythrocyte fragmentation on blood smear, and 4 of them had positive Coombs test results. Therefore, we believed that the positive results of Coombs’ test could not completely exclude MAHA. The lacking of ADAMTS13 has been suggested as an important diagnostic test for TTP [14]. Unfortunately, only 3 patients underwent ADAMTS13 test in this study, and monitoring of the enzyme activity at regular intervals during treatment was not performed. In the future study, ADAMTS13 activity should be monitored, as it may provide helpful guidelines for treatment.

In this study, we found renal impairment occurred frequently, which was in line with the observations in adult TTP-SLE patients. In the study, it was reported that the most common type of renal pathological damage was class IV (57.7%), followed by class V (11.5%), class I (5.8%), and TMA (5.8%) [15]. Patients with severe lupus nephritis usually had TMA observed [9], and the presence of TTP can adversely affect the prognosis of the renal disease [16]. In addition, kidney TMA was usually observed in patients with severe lupus nephritis [9]. In the present study, we also found class IV and TMA the most common pathological damages observed in pediatric patients.

There has been limited consensus on the management for TTP-SLE. Although the mortality rate of the disease could reach as high as 90% if not treated properly, the survival rate has improved to 80–90% since the widespread use of plasma exchange [15]. In addition to plasma exchange, administration of glucocorticoids and immunosuppressive agent were also the treatment options for TTP-SLE. The combined therapies (plasma exchange and immunosuppressor including steroids and cytotoxic agents) were found to be more appropriate than plasma exchange or immunosuppressor alone in treating adult TTP-SLE patients and have shown to achieve a higher remission rate (65.7–90.4% vs 40–50%) [15]. In our research, a 15 years old patient with 13 SLEDAI score who had received glucocorticoid therapy combined with plasma exchange to achieve disease remission, and the number of plasma exchanges was small (4 times). While the combined therapies (plasma exchange and immunosuppressor including steroids and cytotoxic agents) were found to be more appropriate than plasma exchange or immunosuppressor alone in treating adult SLE-TTP [13]. This case suggests that for patients with older age and stable disease, we can try plasma exchange combined with glucocorticoid therapy firstly. and cyclophosphamide was given for all patients received immunosuppressive agent. On the basis of our experience, we suggested that glucocorticoids plus cyclophosphamide could effectively stabilize the SLE-TTP. However, for patients with refractory SLE-TTP, plasma exchange should be performed as soon as possible. Biologic therapy may also be used for refractory SLE-TTP.

Studies showed that rituximab could remove ADAMTS13 inhibitors and improved clinical outcomes of patients with TTP [17, 18]. In this study, the patient administrated with Rituximab showed satisfactory clinical outcome.

The current study has some limitations. First, this is a retrospective study with small sample size. Also, some laboratory and safety follow up data were missing. The results and conclusion thus may not be comprehensive. However, we believe the data reported in this study could provide useful reference for clinical practice.

## Conclusion

Patients with TTP-SLE often had moderate to severe lupus disease activity. Testing of LDH level and blood smear should be performed when kidney and neurological symptoms arise in children with SLE, in order to allow early diagnosis of TTP-SLE. The use of combination therapy, glucocorticoids plus immunosuppressive agent, provided satisfactory clinical outcome; and patients with refractory TTP-SLE need the additional plasma exchange therapy.

## Abbreviations

AIHA: Autoimmune hemolytic anemia
SLE: Systemic lupus erythematosus
SLEDAI: SLE disease activity
TMA: Thrombotic microangiopathy
TTP: Thrombotic thrombocytopenic purpura
